# Cancer caregiving tasks and consequences and their associations with caregiver status and the caregiver’s relationship to the patient: a survey

**DOI:** 10.1186/1471-2407-14-541

**Published:** 2014-07-28

**Authors:** Line Lund, Lone Ross, Morten Aagaard Petersen, Mogens Groenvold

**Affiliations:** The Research Unit, Department of Palliative Medicine, Bispebjerg and Frederiksberg Hospitals and University of Copenhagen, Bispebjerg Bakke 23, DK-2400 Copenhagen, NV Denmark; The Department of Health Services Research, Institute of Public Health, University of Copenhagen, Oster Farimagsgade 5, DK-1014 Copenhagen K, Denmark

**Keywords:** Cancer, Informal caregivers, Cross-sectional questionnaire study, Caregiving tasks, Caregiving consequences, Caregiver status, Caregiver’s relationship to the patient

## Abstract

**Background:**

Seriously ill patients often depend on their informal caregivers to help and support them through the disease course. This study investigated informal cancer caregivers’ experiences of caregiving tasks and consequences and how caregiver status (primary vs. non-primary caregiver) and the caregiver’s relationship to the patient (spouse/partner, etc.) are related to these experiences.

**Methods:**

In a cross-sectional questionnaire study, randomly selected cancer patients with a range of diagnoses and disease stages were invited to pass on the ‘Cancer Caregiving Tasks, Consequences and Needs Questionnaire’ (CaTCoN) to 1–3 of their caregivers.

**Results:**

A total of 590 caregivers related to 415 (55% of 752 eligible) cancer patients participated. Large proportions of caregivers experienced substantial caregiving workload, e.g., provision of psychological support (74%), as well as a range of negative consequences, most commonly stress (59%). Some caregivers experienced personal growth, but relatively large proportions did not. Caregiver status and the caregiver’s relationship to the patient were associated with some caregiving aspects. Primary caregivers experienced the highest caregiving workload, and non-primary caregivers experienced most problems with getting time off from work. Spouses/partners and/or parents experienced the highest workload, most lack of time for social relations, most financial difficulties, and had the greatest need for seeing a psychologist. They furthermore experienced the highest degree of personal growth and had the smallest need for living a normal life while being a caregiver. Yet, regarding the majority of caregiving aspects, no associations with caregiver status or the caregiver’s relationship to the patient were found.

**Conclusions:**

Overall, the findings confirm that cancer caregiving is burdensome. The primary and the closest caregivers seemed to take on most caregiving tasks, but, contrary to expectations, regarding the majority of caregiving consequences non-primary and more distant caregivers were affected to the same degree as the primary and closest caregivers. Initiatives and interventions to support not only the primary caregivers are therefore warranted.

**Electronic supplementary material:**

The online version of this article (doi:10.1186/1471-2407-14-541) contains supplementary material, which is available to authorized users.

## Background

Informal caregivers to patients with a life-threatening disease such as cancer are often deeply involved in the patient’s disease and provide extraordinary and uncompensated care. The caregivers may take on a range of disease related tasks, e.g., provision of emotional support [1–3], physical care [4–7], treatment monitoring [1, 4, 5, 8], and symptom management [1, 5, 8]. In addition, the caregivers also frequently take over or assist with everyday tasks [8], such as cooking [9], housekeeping [4, 5, 7], and child care [9], if the patient has become unable to carry out these [1]. These tasks can be emotionally, physically, socially, and financially demanding [10], and 10–50% of the caregivers experience considerable strain [11, 12]. Negative consequences of caregiving, such as depression [1, 3–6, 10, 13–18], anxiety [1, 4–7, 10, 13–16], distress/stress [3, 7, 13–15, 17], fatigue [1, 6, 7, 15, 16], and insomnia [1, 5–7, 15, 16], have frequently been reported. Thus, caregiving may have significant costs to the caregivers’ own well-being [3, 10]. The strain on the caregiver may also affect the patient [19]. Yet, to take on the responsibility as a caregiver is often regarded an ethical, social, and compassionate obligation and by living up to this obligation and doing ‘the right thing’ , the deed can be motivating and rewarding. Thus, positive consequences of caregiving, such as improved sense of self-worth [5, 13, 17], post-traumatic growth [17], relationship enhancement [20, 21], and altered perspective on living [20], have also been described.

Relatively few large caregiver surveys have been conducted (but see e.g., [8]), i.e., the studies documenting caregiving tasks and consequences have often included relatively small samples of caregivers, many have been qualitative studies, or the studies have elucidated only one or few aspects of being a caregiver. The majority of studies have focused on caregivers with the status as primary caregiver (typically appointed by the patient) or spouses/partners. Not much attention has been paid to non-primary caregivers or caregivers with more distant relationships to the patients than spouses/partners.

The present study is a survey including a large sample of cancer caregivers representing various caregiver status (appointed primary or non-primary caregiver) and formal relationships to the patient (spouses/partners, children, etc.). The questionnaire used is the ‘Cancer Caregiving Tasks, Consequences and Needs Questionnaire’ (CaTCoN) which elucidates a broad range of caregiving aspects. The CaTCoN was developed and validated in order to map the caregiving experience more comprehensively than existing instruments [22].

The aims of this study were:

A) To measure the proportions of cancer caregivers experiencing burdensome caregiving tasks and consequences
B) To investigate how caregiver status and the caregiver’s relationship to the patient are related to the caregiving tasks and consequences.

Regarding aim B, our hypotheses were that primary caregivers and caregivers with the closest relationships to the patient took on more caregiving tasks and experienced more consequences of being a caregiver than non-primary caregivers and caregivers with more distant relationships to the patients, respectively.

## Methods

### Study population and questionnaire distribution

In January-July 2010, a sample of cancer patients was randomly selected from medical records in five hospital departments (departments of oncology, haematology, gynaecology and surgery) at three different hospitals in the Copenhagen area. Non-terminal, adult (18+ years) male and female patients with a range of cancer diagnoses and disease stages were included.

Caregiver questionnaires (three copies) were sent to the patients, asking them to pass the questionnaires on to one to three adult caregivers involved in their disease course. Patients reporting that no caregivers had been involved in their disease course were excluded. The questionnaires were identical except that one questionnaire was directed towards and marked ‘primary caregiver’.

The study complied with the Helsinki II Declaration and was approved by the Danish Data Protection Board (jr.no. BBH-2009-01). The protocol was presented to the National Committee on Health Research Ethics (protocol no. H-1-2010-FSP-22) and was found not to require formal approval.

### Measures

#### Cancer caregiving tasks, consequences and needs questionnaire (CaTCoN)

The 72-item CaTCoN measures cancer caregiving tasks and consequences and the caregivers’ needs mainly concerning information from and communication and contact with health care professionals [22]. The validity and reliability of the CaTCoN were evaluated by using psychometric analyses and tests of convergent/discriminant validity with the existing instruments FAMCARE [23] and FIN [24] and were found to be satisfactory: Cronbachs alpha ranged 0.65-0.95, and hypothesized convergent and divergent CaTCoN and FAMCARE/FIN scales correlated 0.59-0.74 and −0.11-0.25, respectively [25]. The CaTCoN contains nine subscales (each containing between two and 14 items) and 31 single items (including two open-ended items for qualitative comments). The majority of items contain four ordinal response categories and a ‘don’t know/not relevant’ category. Single item scores are expressed on a scale ranging 0 (no burden/problems) to 100 (maximum burden/problems), excluding the ‘don’t know/not relevant’ category. Subscale scores are estimated as the mean of ‘information carrying’ item scores (i.e., responses in all other categories than the ‘don’t know/not relevant’ category) also expressed on a range from 0 (no burden/problems) to 100 (maximum burden/problems). The subscale scores used in the present analyses were calculated if responses to half or less of the items were missing or in the ‘don’t know/not relevant’ category.

The caregivers were also asked a range of socio-demographic questions (see ‘Variables’ section).

### Variables

#### Outcomes

This article reports the results concerning caregiving tasks, i.e., the CaTCoN subscale ‘caregiving workload’ (items 1a, 1b, 1c, 3, 4) and single item 2, and caregiving consequences, i.e., the CaTCoN subscales ‘lack of time for social relations’ (items 6c, 6d) and ‘lack of personal growth’ (items 6e, 6f, 6g), and single items 6a, 6b, 7, 8, 9, 20, 34, 38, 39, 40 and 41 [22, 25] as shown in Table 1, left column.

**Table 1:** **Frequencies (%) and mean scores of responses regarding caregiving tasks and consequences (n = 590 caregivers)**

CaTCoN items								
Frequencies (%)							Mean^a^	Std Dev
**CaTCoN single items**								
***Caregivng tasks***								
To what extent have you had to provide:	*None*	*A little*	*Some*	*A lot*	*Don’t know/not relevant*	*Missing*		
1a. Practical help to the patient?	13	32	24	29	1	1	56.9	34.4
1b. Personal care to the patient?	49	26	12	8	2	3	25.7	32.0
1c. Psychological support to the patient?	3	20	32	42	1	2	71.9	28.7
2. It is the responsibility of the hospitals to make referrals and appointments for examination and treatment. Have you felt that you have been partially responsible for keeping track of whether the patient has been referred and called for examinations and treatments quickly and correctly?	*Not at all*	*To a low degree*	*To some degree*	*To a high degree*	*Don’t know/not relevant*	*Missing*		
	62	16	9	8	4	1	20.8	32.5
3. Have you felt that you have had too much responsibility in relation to home care (personal care, medications, etc.)?	60	20	8	7	5	1	19.7	30.6
4. Have you spent time transporting the patient?	*No, not at all*	*Yes, a little*	*Yes, some*	*Yes, a lot*	*Don’t know/not relevant*	*Missing*		
	23	27	23	25	1	1	50.3	37.0
***Caregiving consequences***								
Has the patient’s cancer disease:	*No, not at all*	*Yes, a little*	*Yes, some*	*Yes, a lot*	*Don’t know/not relevant*	*Missing*		
6a. Caused you stress?	9	29	28	31	2	1	61.0	32.7
6b. Had a negative effect on your own physical health?	52	29	11	5	2	1	22.7	28.9
6c. Meant that you have not had enough time for (the rest of) your family?	49	28	13	6	3	1	25.1	30.5
6d. Meant that you have not had enough time for (the rest of) your friends/acquaintances?	45	28	14	9	3	1	29.1	33.0
6e. Increased you awareness of the important things in life?	5	18	30	45	2	1	27.5	30.0
6 f. Caused you to make positive changes?	19	39	24	11	5	1	57.1	30.7
6 g. Made you value your relationships with other people more?	10	26	31	29	4	1	39.4	32.7
7. Have you been able to take time off, get leave of absence from work, or make similar arrangements to the extent it has been necessary?	*Always/almost always*	*Mostly*	*Only sometimes*	*Rarely/never*	*Don’t know/not relevant*	*Missing*		
	40	13	4	4	38	2	17.3	28.4
8. Has the patient’s illness meant that you have had to be absent from work so much that it has posed problems at your workplace?	*Not at all*	*To a low degree*	*To some degree*	*To a high degree*	*Don’t know/not relevant*	*Missing*		
	*50*	11	4	1	33	1	11.6	22.7
9. Have you experienced negative financial consequences of being a caregiver?	71	11	6	3	8	1	11.6	24.6
20. Have you needed financial counselling?	*No*	*Yes*				*Missing*		
	94	6				0	6.1	24.0
34. Have you needed to see a psychologist as a consequence of the patient’s illness?	80	20				0	20.4	40.3
38. Have you had the need to be able to take a break from the practical tasks (in the role of caregiver) in connection to the illness?	*To a high degree*	*To some degree*	*To a low degree*	*Not at all*	*Don’t know/not relevant*	*Missing*		
	6	16	17	39	21	1	29.0	33.6
39. Have you felt that you have had the possibility to take a break from the practical tasks?	18	18	14	14	35	1	46.4	37.1
40. Have you had the need to lead a ‘normal’ life at the same time as you have been a caregiver?	38	29	12	8	12	1	70.3	32.4
41. Have you felt that you have had the possibility to lead a ‘normal’ life at the same time as being a caregiver?	35	36	16	5	7	1	30.5	29.4
**CatCoN subscales**								
Caregiving workload (items 1a, 1b, 1c, 3, 4)							45.1	23.5
Lack of personal growth (items 6e, 6f, 6g)							41.1	26.1
Lack of time for social relations (items 6c, 6d)							27.3	30.0

^a^Mean score range from 0 (no burden/problems) to 100 (maximum burden/problems).

#### Independent variables

This study focused on two independent variables: 1) ‘caregiver status’, classified as primary or non-primary caregiver to the patient based on the mark on the questionnaire and 2) ‘the caregiver’s relationship to the patient’ , classified as spouse/partner, child, parent, sibling, or other (e.g., friend, colleague) based on information in the questionnaire. Among the latter, we hypothesized that spouses/partners and (for young patients) the patient’s parents would have the closest relationships to the patients.

The following independent variables were also included: *caregiver* gender, age, marital status, having children or not, education, employment, job description, and place of living; and *patient* gender, age, diagnosis, disease stage, inclusion group (i.e., diagnosed within the last year, diagnosed more than a year ago and in treatment, or diagnosed more than a year ago and off treatment), time since diagnosis, and type of hospital department (from which the patient was sampled).

### Data analysis

The proportion of patients with at least one participating caregiver was calculated. Characteristics (shown in Table 2, left column) of eligible patients with at least one participating caregiver and eligible patients without a participating caregiver were compared using logistic regression analysis. Similarly, and regarding the same patient characteristics, the patients with a participating primary caregiver were compared to those without a participating primary caregiver (but with at least one participating non-primary caregiver).

**Table 2:** **Patient characteristics**

		Eligible patients	Patients with participating caregiver(s)	Proportion participating (%)	Odds ratio^#^(OR)	95% confidence interval
**All**		752	415	55		
**Gender**	Female	445	238	53	1.00	-
	Male	307	177	58	1.18	(0.88-1.59)
**Age**	18-39 years	85	43	51	0.88	(0.54-1.45)
	40-49 years	77	40	52	0.93	(0.56-1.56)
	50-59 years	122	71	58	1.20	(0.77-1.86)
	60-69 years	240	129	54	1.00	-
	70+	228	132	58	1.18	(0.82-1.71)
**Diagnosis**	Head and neck	66	37	56	1.11	(0.63-1.97)
	Gastrointestinal	146	85	58	1.22	(0.78-1.89)
	Gynaecological	176	94	53	1.00	-
	Breast	60	28	47	0.76	(0.42-1.37)
	Leukaemia	148	79	53	1.00	(0.64-1.55)
	Other (lung, prostate, urinary etc.)	155	91	59	1.56	(0.83-2.95)
	Missing	1	1	100	-	-
Stage (TNM)**	1	162	76	47	1.00	-
	2	109	48	44	0.89	(0.55-1.45)
	3	79	57	72	2.93	(1.64-5.24)
	4	106	63	59	1.66	(1.01-2.72)
	Cancer with no TNM stage/missing	296	171	58	-	-
Inclusion group*	1 (diagnosed within the last year)	321	187	58	1.42	(1.04-1.94)
	2 (diagnosed > 1 year ago, and in treatment)	96	60	63	1.70	(1.07-2.71)
	3 (diagnosed > 1 ago, and off treatment)	327	162	50	1.00	-
	Missing	8	6	75	-	-
**Time since diagnosis**	< 6 months	179	100	56	0.99	(0.66-1.50)
	6-12 months	138	85	62	1.26	(0.80-1.98)
	1–2 years	120	64	53	0.90	(0.56-1.42)
	2-5 years	182	102	56	1.00	-
	> 5 years	129	61	47	0.70	(0.45-1.11)
	Missing	4	3	75	-	-
**Hospital department**	Oncology/Haematology	569	316	56	1.00	-
	Gynaecology	120	58	48	0.75	(0.51-1.11)
	Surgery	63	41	65	1.49	(0.87-2.57)
Marital status**	Married/cohabiting	294	266	90	1.00	-
	Other (divorced/separated, single, widow(er))	128	91	71	0.26	(0.15-0.45)
	Missing	330	58	18	-	-
**Education**	No education	58	45	78	0.87	(0.39-1.90)
	Student/under education	6	5	83	1.25	(0.14-11.31)
	Non-theoretical or short education (<1 year)	93	82	88	1.86	(0.84-4.14)
	Short theoretical education (1–3 years)	92	84	91	2.63	(1.09-6.30)
	Long theoretical education (>3 years)	100	80	80	1.00	-
	University education	56	49	88	1.75	(0.69-4.44)
	Missing	347	70	20	-	-
**Job description**	Self-employed	53	47	89	1.44	(0.57-3.61)
	Salaried	232	196	84	1.00	-
	Skilled worker	58	49	84	1.00	(0.45-2.21)
	Un-skilled worker	37	30	81	0.79	(0.32-1.93)
	Assisting spouse	9	7	78	0.64	(0.13-3.22)
	Missing	363	86	24	-	-
**Employment before cancer**	Full time	176	153	87	1.00	-
	Part time	45	42	93	2.10	(0.60-7.35)
	Old age pension	119	100	84	0.79	(0.41-1.53)
	Early retirement pension	57	44	77	0.51	(0.24-1.09)
	Other (student, un-employed, housewife)	19	16	84	0.80	(0.22-2.97)
	Missing	336	60	18	-	-
**Current employment**	Full time	124	108	87	1.52	(0.79-2.94)
	Part time	43	40	93	3.01	(0.87-10.37)
	Old age pension	163	133	82	1.00	-
	Early retirement pension	65	55	85	1.24	(0.57-2.71)
	Other (student, un-employed, housewife)	21	17	81	0.96	(0.30-3.06)
	Missing	336	62	18	-	-
**Place of living**	In the country/village	74	67	91	1.64	(0.69-3.89)
	Smaller provincial town	65	51	78	0.62	(0.31-1.26)
	Bigger provincial town	64	53	83	0.83	(0.39-1.75)
	City or suburb	219	187	85	1.00	-
	Missing	330	57	17	-	-

Characteristics (obtained from medical records and self-reported) of the 752 eligible patients and the 415 patients with at least one participating caregiver, and comparison (OR) of characteristics of eligible patients with at least one participating caregiver and eligible patients without a participating caregiver.

^#^Odds for participation is analysed using logistic regression analysis.

*0.05 > p > 0.01 in the logistic regression analysis.

**p < 0.001 in the logistic regression analysis.

The association between caregiver status and the caregiver’s relationship to the patient was investigated. Furthermore, the associations between the independent variables and whether or not the participating spouse/partner was the primary caregiver were investigated using logistic regression analysis.

For the total sample of caregivers, frequencies and mean scores for each of the 22 included CaTCoN items and mean scores for the three subscales were calculated.

The associations between independent variables and the CaTCoN single items and subscales were tested using regression analyses. In the regression analyses, adjustment was made to account for the fact that caregivers related to the same patient were not independent (each patient could have up to three participating caregivers). The adjustment was done by using the PROC MIXED procedure for subscales and the PROC GLIMMIX procedure for single items in the SAS statistical package v. 9.3 [26]. For the linear regression analysis with the three CaTCoN subscales as dependent variables, the normal distribution of the subscale was examined by inspection of residuals, q-q plot, skewness, and kurtosis. To be able to use the PROC GLIMMIX procedure, single items with four ordinal categories were dichotomised in the middle, creating a positive versus a negative response.

In the regression analysis, independent variables associated at a significance level < 0.20 with the particular outcome in an initial univariate regression analysis were entered in a multiple regression model. Backward stepwise regression analysis was undertaken for model building. A significance level of 0.05 was chosen for the multivariate analyses. Only data from the multivariate analyses is shown.

Finally, the association between caregiver status, relationship to the patient, and perceived stress was examined in a three-way table.

## Results

### Study population

A total of 856 patients were identified and were sent questionnaires to pass on to their caregivers. Subsequently, 104 of these patients were excluded from the study as they did not meet the inclusion criteria (e.g., the patients were terminal/deceased or replied that no caregivers had been involved in the disease course). The remaining 752 patients were eligible.

From the 752 eligible patients, 598 caregivers responded to the questionnaire. Of these, eight questionnaires were excluded because the caregiver was <18 years old (n = 5), or the questionnaire was filled in by the patient (n = 3), resulting in a final sample of 590 participating caregivers included in the analyses.

### Characteristics of patients with one or more participating caregivers

The 590 participating caregivers were related to 415 (55%) of the 752 eligible patients as some patients had provided more than one caregiver. Characteristics of eligible patients and ‘participating patients’ (patients with at least one participating caregiver) are shown in Table 2.

‘Participation’ was most common among married patients and among patients with stage three disease and less common among patients diagnosed with cancer more than a year ago and currently off treatment (inclusion group 3) (Table 2).

‘Participating patients’ with a participating primary caregiver did not differ from ‘participating patients’ without a participating primary caregiver (data not shown).

### Characteristics of the participating caregivers

Characteristics of the participating caregivers (n = 590) are shown in Table 3. Concerning caregiver status, about half of the caregivers (51%) were primary caregivers. The caregivers’ relationship to the patients were spouses/partners (46%), children (31%), parents (8%), siblings (7%), and other, e.g., friends or colleagues (8%).

**Table 3:** **Caregiver characteristics**

	n (=590)	%
**Caregiver status**		
Primary caregiver	299	51
Non-primary caregiver	291	49
**Relationship to the patient**		
Spouse/partner	271	46
Child of the patient	182	31
Parent to the patient	47	8
Sibling	39	7
Other (e.g., friend, colleague)	48	8
Missing	3	1
**Gender**		
Female	358	61
Male	228	39
Missing	4	1
**Age (years)**		
18-39	117	19
40-49	120	20
50-59	128	22
60-69	143	24
70+	79	13
Missing	3	1
**Marital status**		
Married/cohabiting	478	81
Other (divorced/separated, single, widow(er))	108	18
Missing	4	1
**Have children**		
No	114	19
Yes	470	80
Missing	6	1
**Place of living**		
Country/village	94	16
Smaller provincial town	97	16
Bigger provincial town	90	15
City or suburbs	301	51
Missing	8	1
**Level of education**		
No education	43	7
Student	18	3
Non-theoretical or short education (<1 year)	104	18
Short theoretical education (1–3 years)	109	18
Long theoretical education (>3 years)	188	32
University education	106	18
Missing	22	4
**Job description**		
Self-employed	56	9
Salaried	345	58
Skilled worker	82	14
Un-skilled worker	40	7
Assisting spouse	12	2
Missing	55	9
**Employment**		
Full time	281	48
Part time	91	15
Old age pension	112	19
Early retirement pension	59	10
Other (student, un-employed, housewife)	37	6
Missing	10	2

Table 4 illustrates the association between caregiver status and the caregiver’s relationship to the patient. Of the patients who had a spouse/partner among their participating caregivers, 64% viewed the spouse/partner as the primary caregiver. Thirty-six percent of the patients with a parent among their participating caregivers viewed the parent as the primary caregiver (of these patients, 88% were below 40 years of age, data not shown).

**Table 4:** **The association between caregiver status and the caregiver’s relationship to the patient**

	Primary caregiver	Non-primary caregiver	Total
**Spouse/partner**	174 (64%)	97 (36%)	271
**Parent**	17 (36%)	30 (64%)	47
**Child**	70 (39%)	112 (62%)	182
**Sibling**	17 (44%)	22 (56%)	39
**Other (friend, colleague, etc.)**	19 (40%)	29 (60%)	48
***Missing***	-	-	3
**Total**	297	290	590

Whether or not the participating spouse/partner was the primary caregiver was significantly associated (p = 0.0351) with the severity of disease: Among patients with stage 1 disease, 77% of the participating spouses/partners were primary caregivers. This proportion dropped to 59% and 56% for patients with stage 2 and 3 disease, respectively, and among patients with stage 4 disease, 49% of the participating spouses/partners were primary caregivers (data not shown).

### Frequency of problems

The distribution of the 590 participating caregivers’ answers to the 22 CaTCoN items is shown in Table 1.

#### Caregiving tasks

Fifty-three percent of the caregivers provided some or a lot of practical help to the patient, 20% provided some or a lot of personal care, and 74% provided some or a lot of psychological support. Although the items indicated that ideally this should not be the case, 17% of the caregivers felt that they to some or to a high degree had been partially responsible for coordinating examinations and treatments, and 15% felt that they to some or to a high degree had had too much responsibility in relation to home care. About half (48%) had spent some or a lot of time transporting the patient.

#### Consequences of caregiving

More than half of the caregivers (59%) reported that the patient’s illness had caused them some or a lot of stress, and 16% reported some or a lot of negative effect on their own physical health. Twenty percent had needed to see a psychologist. Nineteen percent and 23% of the caregivers reported that they had not had enough time for their family and friends, respectively. Twenty-two percent had had the need to take a break from the practical tasks to some or to a high degree, and 67% had had the need to lead a ‘normal’ life while being a caregiver to some or to a high degree. Yet, 28% and 21%, respectively, felt that they had had the possibility for this only to a low degree or not at all.

Eight percent had only sometimes or rarely/never been able to take the necessary time off from work etc., and for 5% the absence from work had caused them problems at their workplace. Nine percent had experienced financial consequences of being a caregiver to some or to a high degree, and 6% had needed financial counselling.

Increased awareness of the important things in life, positive changes, and valuing relationships to other people more was experienced only a little or not at all by 23%, 58%, and 36% of the caregivers, respectively.

### Multivariate analysis

The associations between the CaTCoN outcomes and the two independent variables caregiver status and the caregiver’s relationship to the patient are shown in Table 5 (the associations between the CaTCoN outcomes and the remaining included independent variables can be found in Additional file 1).

**Table 5:** **Associations between CaTCoN outcomes and caregiver status and the caregiver’s relationship to the patient (n = 590)**

	CatCoN outcomes						
	Subscales			Single items			
	Caregiving workload (items 1a, 1b, 1c, 3, 4)	Lack of personal growth (items 6e, 6f, 6g)	Lack of time for social relations (items 6c, 6d)	Problems with getting time off from work (item 7)	Financial difficulties (item 9)	Need for seeing a psychologist (item 34)	Need for living a normal life (item 40)
	Estimate^a^ (SE)	Estimate^a^ (SE)	Estimate^a^ (SE)	OR^a^ (95% CI)	OR^a^ (95% CI)	OR^a^ (95% CI)	OR^a^ (95% CI)
**Caregiver status**	*P = 0.0332*			*P = 0.0224*			
Primary	0 (−)			1.00 (−)			
Not primary	−4.06 (1.89)*			2.23 (1.12-4.41)			
**The caregiver’s relationship to the patient**	*P < 0.0001*	*P = 0.0018*	*P < 0.0001*		*P = 0.0036*	*P = 0.0001*	*P = 0.0116*
Spouse/partner	0 (−)	0 (−)	0 (−)		1.00 (−)	1.00 (−)	1.00 (−)
Child	−12.38 (2.22)**	1.07 (2.68)	−16.23 (3.46)**		0.44 (0.18-1.05)	0.22 (0.11-0.46)	1.74 (0.96-3.14)
Parent	7.01(3.69)	−9.04 (4.14)*	1.76 (4.82)		^b^	1.02 (0.44-2.41)	0.76 (0.33-1.73)
Sibling	−14.67(3.77)**	4.75 (4.35)	−15.51(5.04)**		^b^	0.10 (0.02-0.38)	3.18 (0.97-10.48)
Other (e.g., friend colleague)	−14.56 (3.80)**	13.29 (4.41)**	−11.91 (4.88)*		^b^	0.19 (0.06-0.60)	5.36 (1.45-19.80)
Other^b^ (parent, sibling, friend, colleague etc.)					0.19^b^ (0.07-0.56)		

The associations were tested with multivariate regression analyses also including other independent variables. In this table, only the significant associations are shown (all in all, the associations with 15 outcomes were tested).

^a^A higher estimate indicates higher workload, increased lack of personal growth, and higher degree of insufficient time for social relations, and OR is the odds ratio for ‘problems’/’consequences’.

^b^Item 9 had a quite high number of missings and further a pronounced skewed distribution of answers. To ensure a minimum of three caregivers in each variable response category we collapsed categories in this variable into fewer categories.

*0.05 > p > 0.01, **p < 0.01 in the linear regression analysis of the three subscales.

The status of the caregiver was associated with two out of 15 outcomes. Primary caregivers had higher workload than non-primary caregivers, and non-primary caregivers had more problems with getting time off from work than primary caregivers.

The caregiver’s relationship to the patient was associated with six out of 15 outcomes. Parents to the patient had the highest workload, followed by the spouse/partner. Friends, colleagues etc. experienced the lowest degree of personal growth whereas parents to the patient, followed by the spouse/partner, experienced the highest degree. Parents to the patient, followed by the patient’s spouse/partner, experienced most lack of time for social relations. Spouses/partners experienced most financial difficulties. Parents to the patient, closely followed by the patient’s spouse/partner, had the greatest need for seeing a psychologist. Friends, colleagues etc. had the greatest need for living a normal life while being a caregiver whereas parents to the patient, followed by the spouse/partner, had the smallest need.

Regarding the remaining independent variables (see Additional file 1), caregiver employment and caregiver gender were most often associated with the outcomes. Caregiver employment was associated with five outcomes, but no clear picture emerged regarding which groups were the most burdened. Caregiver gender was associated with four outcomes; women experienced more problems, but also more personal growth in relation to being a caregiver than men.

#### Caregiver status and the caregiver’s relationship to the patient associated with caregiver stress

In line with the multivariate analysis, Table 6 shows that large proportions (47%-71%) of all groups of caregivers, regardless of status and the caregiver’s relationship to the patient, experienced some or a lot of stress. That is, experiencing stress was not limited to the primary caregiver or the caregivers with the closest relationships to the patients.

**Table 6:** **Caregiver stress**

	Primary caregiver	Non-primary caregiver
**Spouse/partner**	110/174 (63%)	63/97 (65%)
**Parent**	12/17 (71%)	20/30 (67%)
**Child**	41/70 (59%)	60/112 (54%)
**Sibling**	10/17 (59%)	11/22 (50%)
**Other (friend, colleague, etc.)**	9/19 (47%)	14/29 (48%)

The association between caregiver status, relationship to the patient, and perceived stress: the proportions (and percentages) of caregivers who felt that the patient’s disease had caused them some or a lot of stress*.*

## Discussion

The present study is unique by including a large sample of caregivers and addressing their caregiving tasks and consequences by using the newly developed and validated questionnaire CaTCoN and by specifically investigating the caregiver status and the caregiver’s relationship to the patient. Most previous studies addressing cancer caregiving have focused on the patient’s partner/spouse (as the primary caregiver) [3, 14] and when caregivers with other relationships to the patient have been included, the associations between these relationships and outcomes have not been examined [18, 27–29]. Also, no previous studies have investigated the caregiver’s status and relationship to the patient in parallel, i.e., examined whether their associations with various outcomes are the same. This study is therefore a valuable supplement to the existing studies of cancer caregiving.

A large proportion of caregivers experienced a considerable caregiving workload related to practical help, psychological support, and transport. Negative consequences of being a caregiver were frequently experienced, e.g., 59% of the caregivers reported stress. Thus, the study documents that being a caregiver is demanding and has its costs; it may jeopardise the caregivers’ own well-being. At the same time, being a caregiver may also bring positive experiences and consequences as seen in this study. However, substantial proportions of the caregivers reported no or minimal positive changes.

Concerning the frequencies of the outcomes in this study, several of the caregiving aspects have been investigated in previous studies, but the findings cannot be compared directly due to the difference in methods. However, the overall findings of this study regarding caregiving workload are in agreement with previous findings. For instance, Yabroff and Kim (2009) found that the time spent by informal caregivers was substantial and burdensome [30], and van Ryn et al. (2011) found that large proportions of caregivers assisted with activities of daily living and moreover watched for treatment side effects (68%), helped managing pain, nausea, or fatigue (47%), etc. [8]. Our finding that distress/stress is commonly experienced by caregivers is also in agreement with previous studies [3, 7, 13–15, 17], but is still remarkable in a country where focus for some time has been on the caregivers and on initiatives to help them.

This study is unique by investigating the extent to which caregiving tasks and consequences vary, depending on caregiver status and relationship to the patient. One might assume that the subdivision of the caregivers into caregiver status and the caregiver’s relationship to the patient would be redundant, expecting that the closest caregivers, especially the spouses/partners (when present), would almost always have the status as primary caregivers. But Table 4 shows that actually more than one third of the participating spouses/partners were not the patients’ primary caregivers, and conversely that a significant proportion of caregivers with the most distant formal relationships as friends, colleagues etc. was in fact primary caregivers. We found that the proportion of primary caregivers among the participating spouses/partners dropped with increasing severity of disease. This could reflect the involvement of several caregivers as the disease progresses. That is, in stage 1, the spouse/partner was often the primary caregiver, and it is likely that no other caregivers were deeply involved in the management of the disease. In metastatic disease, however, more caregivers could be deeply involved in the practical tasks, in the contact with the health care system etc., and perhaps the patient considered all caregivers equally involved (or actually someone else than the spouse/partner as most involved) and passed on the primary caregiver questionnaire to someone else than the spouse.

Regarding caregiver status, one finding in this study was that primary caregivers – as hypothesized – experienced higher caregiving workload than non-primary caregivers. As the primary caregiver by definition is the caregiver most involved in the patient’s disease course, this finding might seem obvious, but this study is in fact the first to focus on and document the association in this way. The other finding was that non-primary caregivers had significantly more problems with getting time off from work than primary caregivers. This finding could seem surprising as the primary caregivers would be likely to have a greater need of getting time off. Yet, perhaps being a primary caregiver entitles the caregiver to make workplace arrangements that enable them to get time off more easily. Caregiver status was not associated with any other outcomes which was contrary to expectations. For instance, it could be reasonable to assume that primary caregivers experienced more stress, more personal growth, more lack of time for social relations, and more negative physical consequences than non-primary caregivers, but neither of these hypotheses were confirmed. These findings point out that in many aspects non-primary caregivers are affected to the same degree as primary caregivers.

Regarding some aspects, the spouses/partners and parents to the patients were most burdened and experienced most consequences of being caregivers. The literature addressing the caregiver’s relationship to the patient is limited, but a few studies have investigated the association between the caregiver’s relationship to the patient and financial difficulties. Yabroff & Kim (2009) and Van Houtven et al. (2010) found that spouses faced higher economic burden than other relatives and friends [30, 31] which is in compliance with our finding. Still, the number of ‘negative’ findings in our study was surprising. For instance, we would have expected that the closest caregivers experienced most stress, but this was not confirmed. In other words, regarding several caregiving aspects, the more distant caregivers were affected to the same degree as the closest caregivers, underlining that focus should be not only on the spouses/partners and parents, but also on the remaining caregivers.

The study had a relatively equal distribution of ‘participating’ very ill and less ill patients: of the patients with participating caregivers who could be assigned a disease stage, 51% had early stages of cancer (TNM stages 1–2), and 49% had advanced cancer (TNM stages 3–4). Also, about half (45%) of the patients with participating caregivers had been diagnosed within the last year (Table 2). It is noteworthy that the patient characteristics stage, time since diagnosis, and in or off treatment were associated with only two outcomes (‘need for living a normal life’ and ‘lack of possibility for living a normal life’) out of 15. That is, the majority of our findings concern not only caregivers to very ill patients or caregivers who has been struggling for the patient for years, but also caregivers to less ill patients and caregivers who recently have become caregivers.

This study shows that there is a need for addressing not only the patient’s primary or closest caregiver which previously has been the norm, but to acknowledge that a patient has more than one caregiver and that they are all affected by the patient’s disease. It is important to acknowledge all caregivers regardless of caregiver status and relationship to the patient and to include them all in studies and interventions. For the future, caregivers should be in plural.

### Strengths and limitations

As in any survey, the generalizability of the findings was limited by non-participation. Nevertheless, as the caregivers were invited by the patients, the fact that caregivers to 55% of the eligible patients responded appears reasonable when taking into account that some patients probably did not report to the study group that they did not have any caregivers sufficiently involved to be considered relevant for the study and instead chose not to respond. Optimally, and to estimate the caregiver response rate more accurately, data regarding how many caregivers each patient had and how many caregivers the patient chose to invite should have been collected.

The fact that the patients were ‘gatekeepers’ for the caregivers may have had an influence on the kind of caregivers invited; for instance, as also described in a recent study of caregivers of Girgis et al. [32], the patients might have wished to spare the most burdened caregivers from more disease related issues and as a consequence have been inclined to invite caregivers with a lower burden. This could be a reason for the relatively large non-response from primary caregivers (i.e., 28% of patients with participating caregivers had no primary caregiver participating), and if this is the case, responses from the most burdened caregivers are lacking in this study, and the results may thereby underestimate the overall level of tasks and consequences among caregivers.

Major strengths of the study are the large sample size, the measurement of a range of aspects regarding tasks and consequences of caregiving, and the elaboration of the associations with caregiver status and the caregiver’s relationship to the patient.

## Conclusions

Our findings confirm that cancer caregiving is burdensome: large proportions of cancer caregivers experienced substantial caregiving workload, such as provision of practical help and emotional support and help with transport. Caregivers reported a range of negative consequences of being a caregiver, most commonly stress. Some caregivers experienced personal growth, but relatively large proportions did not.

The caregiver status was only associated with two outcomes: primary caregivers experienced the highest caregiving workload, and non-primary caregivers had most problems with getting time off from work. Regarding some caregiving aspects, spouses/partners and/or parents (i.e., the caregivers with the closest relationships to the patients) seemed to be the most burdened caregivers, i.e., they experienced the highest workload, most lack of time for social relations, most financial difficulties, and had the greatest need for seeing a psychologist, but they also experienced the highest degree of personal growth and had the smallest need for living a normal life while being a caregiver.

All in all, the primary and the closest caregivers seemed to take on most caregiving tasks, but regarding the majority of caregiving consequences, non-primary and more distant caregivers were affected to the same degree as the primary and closest caregivers. Thus, initiatives and interventions to support all caregivers are warranted.

## Electronic supplementary material

Additional file 1: **Multivariate analysis of the associations between CaTCoN outcomes and independent variables (n = 590).** This file contains the significant results of the multivariate regression analyses of the included 15 CaTCoN outcomes and the included independent variables (except for the variables ‘caregiver status’ and ‘the caregiver’s relationship to the patient’ which have been shown separately in Table 5). (DOCX 31 KB)
