# Supplementary material for: Cancer caregiving tasks and consequences and their associations with caregiver status and the caregiver’s relationship to the patient: a survey
Source: BMC Cancer. 2014 Jul 28;14:541. doi: 10.1186/1471-2407-14-541 (PMC4122762; doi:10.1186/1471-2407-14-541)
Supplement: Supplementary file 1 — Additional file 1: Multivariate analysis of the associations between CaTCoN outcomes and independent variables (n = 590). This file contains the significant results of the multivariate regression analyses of the included 15 CaTCoN outcomes and the included independent variables (except for the variables ‘caregiver status’ and ‘the caregiver’s relationship to the patient’ which have been shown separately in Table 5). (DOCX 31 KB) [file 12885_2014_4719_MOESM1_ESM.docx]

**Additional file 1. Multivariate analysis of the associations between CaTCoN outcomes^#^ and independent variables (n=590).**

|  | **CatCoN subscales** | | | **CaTCoN single items** | | | | | | | | | | | | | | | | | | | | | | | | |  |
| --- | --- | --- | --- | --- | --- | --- | --- | --- | --- | --- | --- | --- | --- | --- | --- | --- | --- | --- | --- | --- | --- | --- | --- | --- | --- | --- | --- | --- | --- |
|  | **Caregiving workload**  **(items 1a, 1b, 1c, 3, 4)** | Lack of personal growth  **(items 6e, 6f, 6g)** | **Lack of time for social relations**  **(items 6c, 6d)** | **Stress**  **(item 6a)** | **Negative physical consequences (item 6b)** | **Problems with getting time off from work**  **(item 7)** | **Financial difficulties**  **(item 9)** | **Need for seeing a psychologist (item 34)** | | **Need for take time off from practical tasks (item 38)** | | | | | **Lack of possibility for taking time off from practical tasks**  **( item 39)** | | | | | **Need for living a normal life**  **(item 40)** | | | | | **Lack of possibility for living a normal life**  **(item 41)** | | | |  |
|  | Estimate^a^  (SE) | Estimate^a^  (SE) | Estimate^a^  (SE) | OR^a^(95%CI) | OR^a^(95%CI) | OR^a(^(95%CI) | OR^a^(95%CI) | OR^a^(95%CI) | | OR^a^(95%CI) | | | | | OR^a^(95%CI) | | | | | OR^a^(95%CI) | | | | | OR^a^(95%CI) | | | |  |
| Caregiver gender |  | *P=0.0203* | *P=0.0065* |  | P=0.0070 |  |  | | P=0.0016 | |  | | | | |  | | | | |  | | | | |  | | | |
| Male |  | 5.38(2.29)* | -7.29(2.64)** |  | 0.48(0.28-0.81) |  |  | | 0.41(0.24-0.71) | |  | | | | |  | | |  | |  | | |  | |  | |  | |
| Female |  | 0(-) | 0(-) |  | 1.00(-) |  |  | | 1.00(-) | |  | | | | |  | | |  | |  | | |  | |  | |  | |
| Caregiver age |  |  | *P=0.0035* |  |  |  |  | | P=0.0097 | | *P=0.0021* | | | | |  | | | | |  | | |  | |  | |  | |
| 18-39 |  |  | 8.72(4.31)* |  |  |  |  | | 3.69(1.46-9.33) | | 2.39(1.30-4.42) | | | | |  | | | | |  | | |  | |  | |  | |
| 40-49 |  |  | 8.69(3.89)* |  |  |  |  | | 1.86(0.79-4.38) | | 0.98(0.51-1.90) | | | | |  | | | | |  | | |  | |  | |  | |
| 50-59 |  |  | 8.54(3.64)* |  |  |  |  | | 1.08(0.47-2.46) | | 1.31(0.70-2.45) | | | | |  | | | | |  | | |  | |  | |  | |
| 60-69 |  |  | 0(-) |  |  |  |  | | 1.00(-) | | 1.00(-) | | | | |  | | | | |  | | |  | |  | |  | |
| 70+ |  |  | -6.01(4.24) |  |  |  |  | | 0.33(0.08-1.31) | | 0.45(0.18-1.13) | | | | |  | | | | |  | | |  | |  | |  | |
| Marital status |  |  |  |  |  |  |  | | *P=0.0172* | |  | | | | |  | | | | |  | | |  | |  | |  | |
| Married |  |  |  |  |  |  |  | | 1.00(-) | |  | | | | |  | | |  | |  | | |  | |  | |  | |
| Other (…) |  |  |  |  |  |  |  | | 2.32(1.16-4.63) | |  | | | | |  | | |  | |  | | |  | |  | |  | |
| **Caregiver education** | *P=0.0158* |  |  |  |  |  |  | |  | |  | | | | |  | | |  | |  | | |  | |  | |  | |
| None | -3.62(3.82) |  |  |  |  |  |  | |  | |  | | | | |  | | |  | |  | | |  | |  | |  | |
| Student | -4.03(5.77) |  |  |  |  |  |  | |  | |  | | | | |  | | |  | |  | | |  | |  | |  | |
| < 1 year or non-theoretical | -0.19(2.78) |  |  |  |  |  |  | |  | |  | | | | |  | | |  | |  | | |  | |  | |  | |
| Theoretical, 1-3 years | -0.32(2.67) |  |  |  |  |  |  | |  | |  | | | | |  | | |  | |  | | |  | |  | |  | |
| Theoretical, > 3 years | 0(-) |  |  |  |  |  |  | |  | |  | | | | |  | | |  | |  | | |  | |  | |  | |
| University education | -9.37(2.71)** |  |  |  |  |  |  | |  | |  | | | | |  | | |  | |  | | |  | |  | |  | |
| **Caregiver employment** |  | *P=0.0101* |  |  | *P=0.0087* |  |  | | P=0.0401 | |  | | | | | *P=0.0414* | | | | | *P=0.0019* | | | | |  | |  | |
| Full time |  | 0(-) |  |  | 1.00(-) |  |  | | 1.00(-) | |  | | | | | 1.00(-) | | | | | 1.00(-) | | | | |  | |  | |
| Part time |  | -3.83(3.14) |  |  | 1.56(0.78-3.11) |  |  | | 0.64(0.32-1.27) | |  | | |  | | 1.51(0.83-2.75) | | | | | 0.44(0.23-0.85) | | | | |  | |  | |
| Old age pension |  | 7.02(3.03)* |  |  | 2.17(1.16-4.05) |  |  | | 0.61(0.19-1.98) | |  | | |  | | 2.82(1.41-5.64) | | | | | 0.43(0.22-0.83) | | | | |  | |  | |
| Early retirement pension |  | -6.30(3.86) |  |  | 3.17(1.55-6.47) |  |  | | 0.66(0.25-1.76) | |  | | |  | | 1.51(0.72-3.19) | | | | | 0.24(0.12-0.52) | | | | |  | |  | |
| Other (student, un-employed, housewife) |  | -2.39(4.49) |  |  | 2.86(1.22-6.71) |  |  | | 2.84(1.22-6.59) | |  | | |  | | 0.75(0.24-2.36) | | | | | 1.21(0.38-3.91) | | | | |  | |  | |
| Patient age |  |  |  | P=0.0026 |  |  | P=0.0027 |  | |  | | |  | |  | | |  | |  | | |  | |  | |  | |  |
| 18-39 |  |  |  | 2.29 (1.17-4.49) |  |  | 10.65(3.16-35.87) |  | |  | | |  | |  | | |  | |  | | |  | |  | |  | |  |
| 40-49 |  |  |  | 1.95(0.98-3.87) |  |  | 4.01(1.20-13.42) |  | |  | | |  | |  | | |  | |  | | |  | |  | |  | |  |
| 50-59 |  |  |  | 1.32(0.77-2.28) |  |  | 2.87(1.06-7.74) |  | |  | | |  | |  | | |  | |  | | |  | |  | |  | |  |
| 60-69 |  |  |  | 1.00(-) |  |  | 1.00(-) |  | |  | | |  | |  | | |  | |  | | |  | |  | |  | |  |
| 70+ |  |  |  | 0.73(0.46-1.15) |  |  | 1.15(0.42-3.21) |  | |  | | |  | |  | | |  | |  | | |  | |  | |  | |  |
| **Patient inclusion group** |  |  |  |  |  |  |  | |  | |  | |  | | |  | |  | | | *P=0.0157* | | | | |  | |  | |
| 1 (diagnosed within the last year) |  |  |  |  |  |  |  | |  | |  | |  | | |  | |  | | | 1.40(0.82-2.39) | | | | |  | |  | |
| 2 (diagnosed > 1 year ago, and in treatment) |  |  |  |  |  |  |  | |  | |  | |  | | |  | |  | | | 3.58(1.49-8.60) | | | | |  | |  | |
| 3 (diagnosed > 1 ago, and off treatment) |  |  |  |  |  |  |  | |  | |  | |  | | |  | |  | | | 1.00(-) | | | | |  | |  | |
| Patient cancer diagnosis |  |  |  |  |  |  | P=0.0190 |  | |  | |  | | |  | |  | | |  | |  | | |  | |  | |  |
| Head and neck |  |  |  |  |  |  | 6.84(1.96-23.88) |  | |  | |  | | |  | |  | | |  | |  | | |  | |  | |  |
| Gastrointestinal |  |  |  |  |  |  | 4.27(1.40-13.02) |  | |  | |  | | |  | |  | | |  | |  | | |  | |  | |  |
| Gynecological |  |  |  |  |  |  | 1.00(-) |  | |  | |  | | |  | |  | | |  | |  | | |  | |  | |  |
| Breast |  |  |  |  |  |  | 2.36(0.56-9.98) |  | |  | |  | | |  | |  | | |  | |  | | |  | |  | |  |
| Leukemia |  |  |  |  |  |  | 1.74(0.55-5.48) |  | |  | |  | | |  | |  | | |  | |  | | |  | |  | |  |
| Other (lung, prostate, urinary etc.) |  |  |  |  |  |  | 1.08(0.33-3.52) |  | |  | |  | | |  | |  | | |  | |  | | |  | |  | |  |
| Patient disease stage (TNM)^b^ |  |  |  |  |  |  |  | |  | |  | |  | | |  | |  | | |  | |  | | | P=0.0256 | | | |
| Stage 1 |  |  |  |  |  |  |  | |  | |  | |  | | |  | |  | | |  | |  | | | 1.00(-) | | | |
| Stage 2 |  |  |  |  |  |  |  | |  | |  | |  | | |  | |  | | |  | |  | | | 1.73(0.76-3.93) | | | |
| Stage 3 |  |  |  |  |  |  |  | |  | |  | |  | | |  | |  | | |  | |  | | | 1.09(0.47-2.52) | | | |
| Stage 4 |  |  |  |  |  |  |  | |  | |  | |  | | |  | |  | | |  | |  | | | 2.90(1.36-6.17) | | | |
| Hospital department |  |  | *P=0.0311* |  |  |  |  | |  | |  | |  | | |  | |  | | | P=0.0198 | | | | |  | |  | |
| Oncology/  Haematology |  |  | 0(-) |  |  |  |  | |  | |  | |  | | |  | |  | | | 1.00(-) | | | | |  | |  | |
| Gynaecology |  |  | -9.95(3.86)* |  |  |  |  | |  | |  | |  | | |  | |  | | | 0.40(0.21-0.78) | | | | |  | |  | |
| Surgery |  |  | -4.76(4.55) |  |  |  |  | |  | |  | |  | | |  | |  | | | 1.15(0.45-2.92) | | | | |  | |  | |

^#^For items 2, 8, and 20, the multivariate regression analyses resulted in no significant associations

a) A higher estimate indicates higher workload, increased lack of personal growth, and higher degree of insufficient time for social relations, and OR is the odds ratio for ‘problems’/’consequences’

b) As not all cancer diseases can be assigned a ’Patient disease stage’, this variable has a substantial number of unknown/missings (n=245), and the analysis therefore included only a subsample. We included this variable in the multivariate analysis if p<0.20 in the univariate analysis, but if the variable was excluded during the multivariate analysis, a new multivariate analysis was done without including the variable in the model (using the whole sample)

* 0.05>p>0.01, ** p<0.01 in the linear regression analysis of the three subscales
